# Blockchains as a means to promote privacy protecting, access availing, incentive increasing, ELSI lessening DNA databases

**DOI:** 10.3389/fdgth.2022.1028249

**Published:** 2023-01-10

**Authors:** Gal Zarchi, Maya Sherman, Omer Gady, Tomer Herzig, Ziv Idan, Dov Greenbaum

**Affiliations:** ^1^ Reichman University (IDC) Herzliya, Herzliya, Tel Aviv District, Israel; ^2^ Zvi Meitar Institute for Legal Implications of Emerging Technologies, Herzliya, Tel Aviv District, Israel; ^3^ Department of Molecular Biophysics and Biochemistry, Yale University, New Haven, NY, United States; ^4^ Harry Radzyner Law School, Reichman University (IDC Herzliya), Herzliya, Israel

**Keywords:** blockchain, ELSI (ethical, legal, and social implications), DNA database, big data, privacy

## Abstract

Not all blockchains are created equal, and many cannot accommodate all of the primary characteristics of big data: Variety, Velocity, Volume and Veracity. Currently, public blockchains are slow and clunky, it can be expensive to keep up with the velocity of genomic data production. Further, the transparent and universally accessible nature of public blockchain doesn't necessarily accommodate all of the variety of sequence data, including very private information. Bespoke private permissioned blockchains, however, can be created to optimally accommodate all of the big data features of genomic data. Further, private permissioned chains can be implemented to both protect the privacy and security of the genetic information therein, while also providing access to researchers. An NFT marketplace associated with that private chain can provide the discretized sale of anonymous and encrypted data sets while also incentivizing individuals to share their data through payments mediated by smart contracts. Private blockchains can provide a transparent chain of custody for each use of the customers' data, and validation that this data is not corrupted. However, even with all of these benefits there remain some concerns with the implementation of this new technology including the ethical, legal and social implications typically associated with DNA databases.

## Introduction

There are millions of human genome sequences (both whole genome and partial) online as a result of various public and private efforts. That number grows daily (1). Online genomic information is increasingly pervasive due in part to the rapid fall in sequencing and storage costs. Concomitantly, direct to consumer (DTC) companies have been selling ever cheaper sequencing opportunities, providing a range of mostly recreational services with varying degrees of scientific validity. Additionally, on the research side, there are many government-funded efforts that collect genomic data from study participants (2–4). One of the largest and most prominent, the All of Us program, is a US government directed personalized health initiative that aims to collect genomic data from up to 1,000,000 US citizens (5).

When the DTC genomics company 23andMe sold access to collected genomic information, they underscored the value of access to these troves of genomic information (6). However, both the potential to deanonymize data to identify private and compromising genetic-based information about people, and the potential to use public genomic databases to also seek out and convict criminals has created a chilling effect on the sharing of genomic data. Many who have submitted or are considering submitting their genetic information to both public and private databases are concerned that their information could be used to put them or a close relative behind bars.

In a worst-case scenario, this expansive amount of publicly accessible DNA information can lead to what the late United States Supreme Court Justice Antonin Scalia referred to as a “genetic panopticon.” (7) Like Jeremy Bentham's proposed prison of constant surveillance, the ability to easily access genetic information that can be relatable to you, either directly or through relatives, creates a reality where our genetic information, when publicly accessible can inform or incriminate anytime and without our consent.

A simple-minded solution would be to store genetic sequencing data offline, encrypted or otherwise in some mostly inaccessible fashion. However, the value of that genetic data to society would be significantly diminished. This dichotomy between simple and easy access to genetic information for research purposes and the need for genetic information to be inaccessible for privacy purposes has long vexed research in this space. Blockchain technology could be part of the solution.

Blockchain platforms are typically immutable ledgers sealed with cryptography and decentralized, with the data siting on thousands if not millions of independent machines or nodes within the blockchain network. Blockchain technology provides a novel storage solution that could allow for continued access to genomic resources while potentially also preventing the misuse of genomic information collected from both governmental and private sources. More than the privacy implications, storing genomic sequences on the blockchain could provide substantial gains in access and usability.

Their potential notwithstanding, there are numerous concerns associated with employing blockchain technology for genomics research. Storing so much data– simply the raw genomic data would comprise at least a gigabyte or more of information– is not necessarily feasible or cost-effective on many public blockchains, like the Bitcoin or Ethereum chains, owning to the massive costs to store so much data. Storing the data off-chain might be more feasible, but then many of the benefits derived from blockchain technology are minimized, such as decentralization and immutability, to degrees.

Nevertheless, in spite of the practical concerns with employing blockchain, in addition to the aforementioned academic research, there are numerous companies that are employing blockchain for genomic and other health-related information (8). Consider, for example, a company like DNAtix which aims to compress DNA sequences by up to 99% of the original size (9), to facilitate the use of public chains. Other companies might consider other possibilities like bespoke private chains. We discuss them herein as well as the ethical, legal and social concerns associated with using either public or private blockchains to store genomic and associated information.

## What is wrong with the current situation?

In the research world, databases for genomic information have goals that may sometimes come into conflict. Researchers need data to be accessible, transparent, reliable and informative and standardized. On the other hand, data is often private and revealing and needs to be inaccessible to those lacking the required permissions. Even those that have permission, may need different versions and different accessibility of the data, depending on many factors, including the nature of their research. And there is always the concern that data, even if permission is legitimately granted, can be misunderstood and misused, or leaked.

Individuals who sequence their genome either for research or for recreational uses are often keen to learn about the results of that research. In other circumstances, sequenced individuals do not want to learn about the outcomes of relevant research, particularly if there isn't anything that can be done to ameliorate the future concern. In some situations, the researchers arguably have an ethical obligation to inform sequenced individuals of actionable medical information gleaned from the data, in some situations they might have an ethical obligation not to inform the sequenced individual, and in some situations, they may have an obligation to inform the extended family of the individual. In addition to the enormous ethical burden, the administrative costs of tracking those that want to, need to, or don't want to be informed of findings can be overwhelming, especially for smaller research endeavors. Further complications can arise when sequenced individuals will allow research to be conducted on their genomes in some areas, but not others. This limitation can complicate access to data when informed consent is required for each new research direction. All of this can inhibit sharing and access to data.

Other concerns associated with standard genetic databases include: (i) data corruption or database failures can limit the usability and reliability of data; (ii) access to DNA databases can be expensive, and researchers can benefit from reliable access that is priced according to their needs; and, (iii) ostensibly anonymized DNA and its associated private information can be accessed without an individual's consent even though that information can often be deanonymized. There remains a fundamental question as to whether a DNA sequence can ever remain truly anonymous; studies have shown that even purportedly anonymous genomes can allow, at least to some extent, the determination of the donor's identity (10–12).

The law often moves glacially slow in response to evolving technologies. Laws and regulations lag behind the innovations in DNA sequencing and analysis (13) and any current enforcement of privacy policies and protection of DNA data are still in their developmental phases.

In summary standard DNA databases have been unable to deal adequately with all of these and other governance issues (14). As such, a technological solution that can provide usable access without impinging on the sequenced individual's ethical rights is needed. That technical solution can be found in blockchain technology.

## Blockchains

Since time immemorial ledgers have formed the backbone of most economies, recording contracts and payments for buying and selling of goods or the exchange of assets. These ledgers started out as records on clay tablets, later on paper, ultimately forming the books supporting modern accounting. Over the last decades these records have moved into the digital realm which made the current complex global economic system possible. Innovation in data keeping continues even today as ledgers are shifting to a global network of computers which is cryptographically secured and decentralized commonly known as a distributed ledger technology (DLT) or blockchain technology. The pseudoanonymous Satoshi Nakamoto described and created the first model of the blockchain (15).

Blockchain can be understood as a decentralized distributed digital database. Until recently, digital databases were designed to centralize information (16). The blockchain though, uses a network of independent computers to maintain a shared database across many nodes (17). Succinctly: when new entries into the database are made, they are automatically broadcast across the blockchain's decentralized network, creating redundant and exact copies. Blockchains claim to be trusted databases, with this trust maintained by open secure computer code and encryption running on all of the decentralized and distributed nodes.

More technically, a blockchain is a set of agreed upon protocols and cryptographic methods that enable a network of computers to work together to securely record data on a shared open database. Every transaction of information is registered and timestamped; all other participants can see that registration. Metaphorically, a blockchain may be considered as a series of blocks of data that are securely chained together. The chain of blocks are linked and secured using cryptography (18). New blocks within a blockchain are formed as contributors create new data. These blocks are encrypted and given a hash value that represents a unique identifier of the data within that block (19). This hashing works *via* a standard algorithm being run over the block's data to compress it into an alphanumeric string. This hash value can be recalculated from the underlining file, confirming that the original contents have not changed. Any minor change within the data will result in a substantially different alphanumeric string—but the reverse is not possible: given just the hash value you cannot recreate the data contained in the block.

All blocks of data which are formed after the first block are securely chained to the previous one *via* the hashing system. Thus, once recorded, the data in any given block cannot be altered afterwards without the alteration of all subsequent blocks across a majority of the identical blocks residing across all the nodes within the network. This hashing and linking of blocks make canonical blockchains resistant to modification of their data. The records are effectively immutable as they cannot be deleted or changed without a consensus of the majority of nodes within the change. Without this majority agreement, a consensus algorithm runs across all nodes hosting the blockchain to make sure that there are no lone outlier records within the network that do not match other versions within the network, for example, due to data corruption. As such, data stored on the blockchain is generally considered incorruptible.

Another principle characteristic of canonical blockchains is that they are distributed systems. This means there is no centralized organization to maintain and verify the entries on the database. Instead, this database is maintained by a large number of computers that are, in some blockchain systems, incentivized to provide computing resources by earnings some form of tokens in exchange. Any computer that is connected to the blockchain network can perform the task of validating the transactions taking place in the network. Validated information is ultimately saved on all the computers nodes in the network providing a greater degree of reliability and protection against data corruption: While each computer node in the network cannot be unwaveringly trusted individually, the system provides a mechanism for creating consensus between the distributed nodes, resulting in the necessary trust and reliability.

### Immutable database—tamper proof

To successfully and intentionally tamper with the blockchain you would need to alter all the encrypted blocks on the chain going forward from the one you altered so that all the aforementioned hashes reflect the underlying data. In a true distributed public chain this would require that the interlocuter take control of more than 50% of the peer-to-peer network. Only then, when more than half of the participants in the network have the altered record will it become the consensus of the chain. On a blockchain of almost any substantive size this would be difficult to do. On larger worldwide networks it is effectively impossible. Of course, this only protects on-chain data. Storing information off the chain and linking to an on-chain identifier will not protect that off-chain data from bad actors or data corruption. Further, on chain data is only truthful with regard to the time of entry and in some specialized cases like the bitcoin blockchain, the number of bitcoins owned by the various users. The blockchain, however does not attest to the veracity of the underlying data—i.e., anyone can easily submit false data to the blockchain, as there are no barriers to this.

### Blockchain taxonomy: public and private blockchains/permissioned and permissionless blockchains

While blockchain technology was initially introduced to the world as a mechanism to enable Bitcoin, it has become increasingly recognized that this system is secure enough to work as a ledger for limited access databases for governments and for private financial institutions. To accommodate the varied institutions that use blockchains there are numerous types of blockchains. These include permissioned and permissionless and private and public chains. Most recognize the public blockchain versions, such as the Bitcoin and Ethereum blockchains. These are ostensibly ownerless entities run by consensus of the nodes. There is no centralized or trusted authority. These chains are also permissionless. In permissionless blockchains, anyone can download the necessary software, become a node within the blockchain and have access to all of the canonically transparent information. Nodes can read, write and/or audit the blocks of data. In public blockchains, in principle, any of the nodes can be the originator of the information in the block.

Permissionless chains are often public and privately owned chains are often permissioned, but that needn't be the case.

Nodes in permissionless blockchains are typically anonymous or at least pseudonymous. These types of blockchains stake their trustworthiness and reliability on the hope and belief that colluding bad actors control less than 50% of the nodes. Any changes to the blockchain software protocols are consensus driven.

Public blockchains lack a trusted intermediary by design and rely on their consensus and validation software run over numerous independent nodes for validation. That validation can sometimes be expensive and energetically wasteful depending on the type of validations used. One prominent type of validation is proof of work. This validation stems in part from miners that compete for fees, that fee is funded by those completing transactions on the chain. At the time of writing, the Ethereum public permissionless blockchain only just implemented software changes that will switch the chain from proof of work to proof of stake. Whether this will significantly increase the speed of transactions, reduce the cost of using the Ethereum platform, and reduce its environmental impact remains to be seen (20).

Permissioned chains are closed to only those nodes that are granted permission through invitation. Private and permissioned blockchains are often used for enterprise applications and are often much faster and more efficient than public and permissionless chains. Private blockchains can employ a trusted intermediary to mediate the operation of the network, which is often considerably smaller than public networks, although private chains can also incorporate mining operations for validation. Private and permissioned blockchains, their centralized authorities notwithstanding, still often operate on decentralized distributed ledger system. In some cases, the centralized authority may maintain a primary copy of the ledger and permissioned users can set up nodes with access to some but not necessarily all of the ledger data.

While all nodes are typically equal in permissionless chains, in a permissioned chain or in a private chain, some nodes may have more rights and capabilities than others; the central authority can choose the degree and nature of transparency for the data for each user. Private and permissioned chains can also be run by a closed consortium wherein some or all the members themselves represent the trusted intermediary. Hybrids of the permissioned and permissionless blockchains also exist. They may be relatively open in terms of the ability for the public to become a node, but they have a trusted intermediary in a central authority.

## Genetic information on blockchains

Given the concerns and problems associated with standard DNA databases described above, there are many advantages to saving and storing genomic information on a blockchain. This paper will focus principally on private/permissioned blockchains as public blockchains raise their own difficult privacy concerns given their transparent nature. They are also typically slow and expensive and the amount of data that can be stored on the chain is inherently restricted; they are not necessarily better DNA data storage options than standard databases.

Private permissioned blockchains on the other hand can be constructed such that they can host large amounts of data on chain rather then simply pointing to the data that resides elsewhere. Hosting the data on chain can allow for greater security and reliability: with multiple copies of the database hosted in various nodes, the chain can rely on a consensus algorithm to maintain a dataset that reflects the majority of nodes and not the minority of corrupted nodes. Versions of private chains for storing genetic information have already been described (21).

A private blockchain further allows for data to be stored safely and securely while still allowing for infinitely-discriminating access to permissioned users. Each user can be granted their own tailored access to the data in terms of things like timeframe of use, nature of the data provided, amount of the data requested, off-chain usability of the data and more. Moreover, private chains relying on a trusted intermediary don't necessarily require the slow and expensive validation steps of public blockchains. Their software protocols are also easily adjustable and adaptable by a central authority when necessary.

A private blockchain can be designed with the specific needs of the genomics research community. For example, each block can represent a single individual and all the associated data, including genomic and phenotypic information. Alternatively, data for a single induvial can reside on multiple blocks, as its added, and yet still be associated with the sequenced individual *via* a unique identifier stored either in the chain, or off chain. The second option allows for updates and additions to the data while still maintaining cryptographically ensured immutability of the data.

Permissioned users can be allowed to either add and/or view data on the chain depending on the centralized authority. Reading, writing, and accessing of the data can be recorded and cryptographically timestamped within the blockchain itself providing a more reliable chain of custody of the data as well as keeping track of who uses and submits data to the chain.

## An NFT marketplace for genomic data

Consider the following technical solution for empowerment of sequenced individuals while creating access to data for researchers.

Each sequenced individual is indexed within the blockchain *via* a unique identifier. Their genomes and medical information that reside throughout the private blockchain, added periodically as more data becomes available, are all tied to that identifier. At least one non-fungible token (NFT)–a unique, often standardized cryptographic asset associated with data on the blockchain, comprising some data or metadata—for each sequenced user is created to transparently represent each individual identifier within the database. That NFT can be anonymous or not, depending on the database or the sequenced individual, although to protect the genomics of the extended family the NFT ought to be anonymized, keeping in mind that at minimum, the NFT will be associated with an IP address or a wallet, likely the wallet or IP address of the sequenced individual.

More broadly, NFTs were initially designed to create scarcity where there was none. Consider digital art online. In the non-digital art market, value is partially associated with the rarity of the art. With online art there is no scarcity as the art can usually be reproduced infinitely, often without the permission of the artist, with no noticeable loss of quality. Consider the canonical example of an image that has been online for years, copied thousands if not hundreds of thousands of times. In these cases, a digital artist is faced with the possibility that collectors will not pay for this art if it is freely available online to everyone else. The NFT was created to artificially create the scarcity component for art. While there may be millions of copies of the art, there is only one (or more) unique, non fungible tokens associated with the art. The NFT is linked *via* the coded smart contract within the NFT to said art by way of a unique identifier.

The NFTs need not confer any actual rights vis-à-vis the original digital art, or any item, digital or corporal, for that matter, that is linked to the NFT. Rather the NFT represents only a unique link to that object. Moreover, they need not confer any ownership rights over an image or digital object that are often otherwise continued to be freely copied and used online.

More simplistically, the NFT is a cryptographic token that represents some relationship between the owner of the token and the item that the token represents. Arguably, an NFT is metaphorically an authenticated signature associated with a piece of art.

More technically, NFTs are smart contracts (specifically built upon the ERC-721 standard that dictates details within the contract such as ownership security and other metadata) that represent a bundle of ownership rights associated with an object, typically a digital object and often a piece of art, although NFTs can be employed to tokenize ownership of art and real-estate in the non-digital world. Rarely do NFTs confer undiluted ownership, and often NFTs will retain ownership rights for the creator of the NFT, e.g., the artists, such as pass-through royalties.

While NFTs as a collectable or investment product have seen their market erode substantially (22), NFTs remain valuable tools to identify and track information as it is traded among the blockchain. NFTs need not contain the information they represent, only code that can be used to identify and locate the information or digital file.

In our case, the NFT is associated with genetic, phenotypic and/or medical data. The token is a unique connection to that data, and the holder of the token can be granted some rights in that data, including access and manipulation. Moreover, the flexibility of the NFT smart contract standard allows the incorporation of other data within the NFT associated with genomic information. This data can include information regarding a user's privacy preferences, research-area preferences, informed consent and other relevant legal information. Alternatively, rather than storing this information on chain in the NFT, it can also be stored off the blockchain, albeit associated with the unique identifier within the NFT.

Off chain data can be stored on distributed files systems like the IPFS, the interplanetary file system or centralized databases. Notably, off chain data can also be more easily modified than data in an NFT, as might be necessary with regard to evolving preferences and informed consents. However, at minimum, the computer code residing within the NFT should provide anonymized basic relevant information about the sequenced user, including information like gender, basic medical history including disease, and other information that might be important to a biomedical researcher seeking out sequences to include in their research. Regardless of what data is stored in the NFT, the NFT will spell out the immutable connection between the genomic and medical data and the metadata on the NFT.

Researchers keen on researching a particular disease or condition, for example, can purchase the relevant NFTs on a purpose-built genomic research marketplace. The purchasing of the NFT would trigger a smart contract that would provide the researcher with access to genomic sequence data, demographic and health data of the individual. That data can be unencrypted, or even encrypted, perhaps employing asymmetric encryption techniques to grant that access.

Each NFT can be designed to create limitations associated with the sale of the information, including limits on using the data relating to time, or nature of the research. The smart contract could also automatically send a percentage of the sale price of the NFT to the sequenced individual. In this way, not only would the sequenced individual be compensated for their data, they would also have an idea as to who is using their data and for what purposes. The marketplace as well as the centralized trusted intermediary would also have this information. The centralized trusted intermediary would also have the ability to deanonymize the sequenced individual as well as restrict or open up access to the said sequenced individual.

NFTs could also be created by the owners of the private blockchain to reflect aggregated sequence information, such that an NFT could be purchased by a researcher that would provide access to all genomes identified within the blockchain containing a particular sequence at a particular locus, or particular expression data for a particular gene. In this way, researches can gain access to a limited but relevant dataset. These transactions as to what data was obtained for what purported use at a particular time can all be recorded on or off the blockchain. Those who wished to revisit and validate research could simply call up the same NFTs purchased by the original researcher with the exact information and redo the analysis. These NFTs could be created ex nihilo to reflect the nature of the research, e.g., the research queries, being performed on the genetic and phenotypic data in the database.

Sequenced individuals could even set the price for their associated NFTs reflecting their actual desire to be part of research. Market forces would likely drive down the average costs of those NFTs. Users could also set different prices for their NFTs depending on the public or private nature of the research, perhaps making academic research more affordable than commercial research. Knowing that their data was included within a particular study, the sequenced individuals could also follow up on the research to seek out actionable information, if any. Each transaction with their NFT would be immutably recorded on the blockchain.

This NFT marketplace could be restricted to qualified individuals and institutions so purchasers of the NFTs would not be able to be anonymous. Similarly, secondary sales of the NFTs would have to be limited if not outright prohibited, as there would be concerns that data would be used by secondary non-qualified researchers. The NFT code could also include time limits on the use of the data to prevent subsequent unauthorized misuse, as well as restrictions as to which IP addresses could access the data represented by the NFT. The system could also use IP addresses to automatically provide cheaper pricing to institutions in developing nations or to IP addresses associated with educational research institutions.

In a more ambitious project, sequenced individuals themselves could use their own NFTs to run analyses on their data. Thus, as a further incentive to provide their data to this secured system, individuals could access various programs and applications that would run analyses on the data. Much of these analyses would have to be recreational in scope given concerns of misinforming the public, but an individual could also use their NFT associated with their genomic data to grant access to their physician to assess the genome for more medically actionable information.

NFTs need not be the only tokens associated with this database endeavor. Just as bitcoins are tokens granted to bitcoin blockchain miners, the private blockchain could incentivize miners within their system as well. Tokens would be granted in exchange for validation. In this case, the token represents the value of accessibility to the genomic data in the database. The tokens can be provided in exchange for access rights on the blockchain, including storage, data access, and information access. These tokens provide both utility as well as a potential investment to support the private chain endeavor.

Regardless as to how access to a private blockchain is mediated, the technology can allow for limited and controlled access of genomic and associated medical and demographic data that is reliable and distributed amongst many nodes, preserving accuracy and accessibility. However, when implementing blockchain technology for genomic sequence information we need to be cognizant of the many potential, ethical, legal and social considerations. Some of these issues are generalizable to all types of databases, not necessarily blockchain. We will endeavor to cover those issues specific to blockchain.

## Similar efforts to employ blockchain and NFT technology for genomics research

Numerous papers have suggested that blockchain technology can provide some of the solution (23). Our solution however is a bit different. Consider for example Musamih et al. (24). The paper provides a broad description of the various uses of NFTs in healthcare, including the ability to employ encryption to help deal with privacy concerns. For example, the paper discusses issues relating to digital twins in healthcare, which we have discussed earlier, here (25), here (26), and here (27). The paper also suggests saving data both on and off chain as well as employing private chains, which we suggest employing as well. However, the paper suggests that control of the data be relegated to the patient. We disagree. While the patient can price the NFT to limit access, or can include limitations on the use of the data, once a researcher purchases access to the patient's data, they have control, as described herein. Further, the paper describes full access to the metadata of the blockchain with regard to the NFT. Here we suggest that in private chain, the patient can choose who can access that metadata and to what degree. Additionally, in Musamih et al., the authors suggest that the patient need not monetize the NFT. We disagree. Each use of the NFT ought to be charged, if for no other reason then that the fees that the NFT exchange collects can be used to help fund the entire endeavor. Allowing some NFTs to not charge for access might also skew research toward those genomes that are free to access. This might further limit access to minorities and underrepresented populations who historically might be poorer and need to monetize their NFTs whereas wealthier and perhaps non-underrepresented populations might need the funds less/ This can bias the data. As such, in our proposal, we suggest that it is best to set a minimum proposed default fee per NFT that we hope all users will use, to reduce bias.

Another similar effort describes the company Genobank.io (28). As per their paper and their website, Genobank will employ end-to-end encryption on the biosample and test results flow both to and from the patient. Genobank similarly claims to be employing a private blockchain, albeit one that is decentralized and immutable. As we discuss herein, we suggest that both or these classical characteristics of blockchains may not be best for the genomic and healthcare records themselves, as this data can be sometimes variable and/or may need to be updated to reflect new and/or better data collection. Genobank also aims to have the genomic data stored in a cryptocurrency wallet, i.e., off-chain. We discuss both the pros and cons of saving data off chain and support both possibilities on a private blockchain. Moreover, as per Genobank's description of their services, the data seems to be held locally by the patient. We disagree with this system as it creates inefficiencies in the process; or system has the data help centrally either within the blockchain or off-chain, but associated with data on the blockchain. This will provide greater efficiencies, especially dealing with the potentially millions, of genomes that could be stored within the system. Finally, the paper focusses on the privacy policies under CCPA, the California Consumer Privacy Act (29). Subsequent to the paper's publishing, California signed the Genetic Information Privacy Act into law. The law took effect in January 2022 and was intended in part to provide particular legislation for genomic privacy. GIPA is directed toward direct to consumer (DTC) genetic testing companies. Given this limited focus, its not clear that all data stored in our proposed platform would qualify for GIPA protection, and as an extension, CCPA, this will especially be the case in a truly decentralized system where no one entity can be found to be liable for privacy infringements.

## Technical implementation of the blockchain for various potential user groups

Privacy is of utmost importance with regard to genomics and health care records, especially, keeping in mind that the disclosure of an individual's genomic data has implications for that individual's immediate and even extended family (30). To some degree, private corporations have already begun to consider the privacy aspects relating to putting genomes on the blockchain (31).

In the proposed platform, we envision at least three potential distinct user groups: (i) patients, their doctors and their families; (ii)researchers, both academic and industry, and, (iii) to a very limiting degree, the general public. Each of these groups are accessing the platform for different needs and purposes and will be availed different opportunities to use the platform depending on those needs and purposes, potentially at different price points depending on characteristics like geographical location and nature of the institution accessing the data.

However, ultimately the goal is to provide the most efficient usability whilst still endeavoring to protect the privacy of the data in the platform for each user group. In addition to providing usability, efficiency and privacy, however, the system is further intended to provide incentives to the first group, the patient group, to submit as much data as possible to the database, as the more data that is provided the more useful the database is to researchers. In this case, while we have described the use of NFTs to provide a monetary incentive, others have suggested some form of security or other representative financial instrument within the larger database as an incentive for populating the database (32).

Public permissionless blockchains are not necessarily designed to provided discriminating access to data; defining characteristics of a public permissionless chain are transparency and accessibility. As such, with each node gaining access to the entire dataset, and with no barriers to entry to become a node, the privacy of an individual within such a blockchain cannot be sufficiently protected unless all the data is encrypted. As noted prior, encrypting the data limits useability of the data.

Thus, recall that our proposed blockchain platform is a private permissioned one. As such, a trusted intermediary can designate the nature and amount of data that can be provided to each user group, as well as the amount and nature of the data that will be accessible to the various nodes supporting the decentralized database. For example, while a researcher (group II) may have access to anonymized data of each individual in the dataset, only a patient (group I) or their physician or their family can access actual names of relatives represented in the patient history. Groups II and II might have access to thousands if not hundreds of thousands of individuals within the dataset, however, Group I would only have access to their own data. Another example, Group III, the general public, including perhaps law enforcement upon production of a warrant, or professional genealogists, might be granted access to the genomic data, and some demographic data, but not the medical and clinical history data.

To further protect the privacy of the individuals in the dataset, the data held in the blockchain can be encrypted, such that even an inadvertent disclosure to an unauthorized individual will still not disclose personal and private information regarding an individual in the dataset. In some cases, one could imagine that the researchers and the public (Groups II and III) might be able to analyze the data *via* homomorphic encryption without the necessary step of decrypting the data and exposing personal and private information (33, 34). Group I, patient, doctor or family might have access to unencrypted data.

Through their minting of NFTs representing their data, the patient can also choose how much information they are willing to share with various group, keeping in mind that that the NFT marketplace that assesses the value of the NFT representing the patient's file will value a more in-depth file. Still, it remains up to the patient to decide on the level of data that they wish to share with other groups accessing the database. More specifically, a patient could mint different NFTs each representing different amounts of data that they are willing to share with third parties.

The trusted intermediary may also include more ethical concerns within their control over genomic data. For example, a patient might be unable to adequately process statistical information relating to their disease risks. In these cases, the trusted intermediary might even limit the type of data that the patient can access given concerns that they might mismanage their own information and make drastic life choices based on misunderstood genomic information (35).

Alternatively, in these cases the data might be released to the patient only through a verified physician or trained genetic therapist. Similarly, a patient may have sequenced and submitted their genomic data for a particular purpose and researchers may have stumbled across an actionable incidental finding, that data might be shared, *via* the blockchain system with the patient's physician, perhaps even automatically *via* a smart contract. At this point it would up to the physician to decide how the information might be best shared, if at all, with the patient and/or their family.

Additional data that might be stored on or off the blockchain and might be made available only to the patient could include a list of the researchers who have gained access to the patient's data and the outcomes, if any of any research done on that data. This could include metadata from the NFT marketplace, describing the type of research done on the datasets.

Typically, all users of an NFT marketplace have access to the historical data associated with the NFT, including past sales. However, as this data could be construed as private –small directed studies that include the data could indicate that there is relevant information related to the disease being studied within that file— the trusted intermediary may by default limit access to this type of metadata. Alternatively, a patient who provides their data and mints an NFT from their clinical and genomic data can request that the transactional history of their NFT, including all potentially privacy infringing metadata be masked from the marketplace.

## Information rights

While information rights are relevant for all DNA sequences regardless as to the nature of the database that stores them, storing DNA sequences on a blockchain raises some specific concerns. Individuals who are sequenced arguably have the right to know their genomic sequence, and arguably, the right to limit the use of their information. DNA sequence data should be as accessible and transparent as possible for the individual, and practically, for researchers as well. However, the sequenced individual's right, which stems from their autonomy, is often not absolute. Both in the UK and Israel the law limits an individual's right to know their genetic data by granting medical practitioners the discretion whether to reveal DNA test results in some situations.

Alternatively, in some cases, the right to know one's genetic information is broad, going beyond the sequenced individual. Relatives arguably also have some right to access relevant information culled from the genome of the sequenced individual (36), given the amount of DNA that they share. For example, identical twins share effectively 100 percent of their DNA, parents and siblings share up to 61 percent of their DNA, and even distant third cousins share up to 2.2 percent of their DNA (37). Thus, when one family member undergoes genetic sequencing and analyzing this has an impact and consequences on the rest of his family, even distant cousins (38).

The sequenced individual similarly may have a right to not disclose their sequenced information. The right not to know has been explicitly recognized: Article 10.2 of the European Convention on Human Rights and Biomedicine states: “Everyone is entitled to know any information collected about his or her health. However, the wishes of individuals not to be so informed shall be observed”. The Explanatory Report to the Convention justifies the right not to know by saying that “patients may have their own reasons for not wishing to know about certain aspects of their health” (39). Similarly, the UNESCO Universal Declaration on the Human Genome provides (Article 5c) that: “The right of every individual to decide whether or not to be informed of the results of genetic examination and the resulting consequences should be respected.” (40) Although some have suggested that this right is limited to the context of the doctor-patient relationship (41).

There can also be duties not to disclose information: Genetic information is often complex and the connections between genetic sequences and disease states is often not straightforward or simple. This can confuse individuals leading to situations known colloquially as the worried well and the walking sick—essentially misunderstanding genetic information can cause an induvial to over-or-under assess the severity of the link between their genetic sequence and a disease. To wit, in Israel, the Genetic Information Law Article 10 rules that only a genetic counselor or an otherwise qualified individual is authorized to give genetic tests results.

With all of these privacy and access limitation concerns, its clear that storing genomic information on a permissionless public blockchain where data can be accessed by anyone all the time can be problematic. On private permissioned chains however, this is of lesser concern. In the example described above, the private chain can be built such that access rights to data can be easily allowed and just as easily revoked. Under the guidance of an ethical committee, the central authority can also decide who can and who cannot gain access to the chain itself, or more specifically to specific sequences.

## Information ownership

The issue of ownership of genetic information is perhaps easier to determine with data stored on public blockchains in contrast to private chains. Individual users who store their data *via* public chains arguably are the owners of their own data, as by design, no one owns a public blockchain and no one else can claim ownership or even copyright rights under a theory of compiliations of database copyright. And while public chains are often too expensive and too restrictive to store genomic sequences, they can still be employed to identify and transact information that is stored off-chain in things like distributed databases. Sequenced individuals can store their data in this fashion and even create their own NFTs to share their genomic data, with or without any limitations. Private chains on the other hand are more like private databases, and the issues regarding DNA ownership in private databases are the same whether the database is a distributed ledger or a single excel file on a single computer. Raw genomic data is often argued to belong to the sequenced individual, its not necessarily the case, however for processed information. The ownership question is also relevant for the outputs of genomic research. While researchers arguably own their resulting research, it could be argued that the sequenced individual retains some rights to even the research outputs. In the example described above, any rights that a sequenced individual might claim should be spelled out in smart contracts associated with the transaction. Users who retain too many rights will likely see that their data remains unused.

## Information veracity & stewardship

In a decentralized public blockchain the software underlying the chain is designed to be responsible for validating that the data remains uncorrupted. Although once in a blockchain, data is less able to be tampered with, blockchain technology does not natively provide any ability to validate that the data that is created outside the system and then entered into the system is itself reliable. A centralized authority of a private chain could conceivably implement safeguards to police and prevent false information from getting onto the chain, this might be more difficult in a public chain, especially one that is not purpose-built for storing genomic information. In a public chain, liability for error that creeps into the data, for example suggesting that an individual does or does not have a genetic condition, cannot be easily assigned, as there is ostensibly no owner or anyone that can held responsible. On private or permissioned chains, negligence can be assigned to the centralized authority that manages the chain.

Another veracity concern relates specifically to public blockchains wherein anyone could conceivably add their data to the distributed ledger. Unfortunately, without oversight and a centralized system there is no way to confirm that the data was transcribed accurately, or if even the data is legitimate. Moreover, malicious users can upload malware to a public chain hiding within the genomic data that could infect systems using the data. This is a cyberbiosecurity concern (42). Both public and private chains could conceivably run software on their ledgers to make sure that uploaded information isn't clearly infectious code. Further, on bespoke systems designed as a genomic database, the centralized authority could also review the genomic data itself on the system to confirm that the genetic information is what it claims to be and not a problematic sequence that could, if printed as a gene, create havoc (43).

## The right to be forgotten

Under the General Data Protection Regulations (GDPR) there is an increasing awareness relating to privacy issues on the internet, including a somewhat novel right to be forgotten. Although not absolute, Article 17 of the GDPR entitles the data subject, e.g., the sequenced individual, to have the data controller erase their personal data, cease further dissemination of the data, and potentially have third parties halt processing of the data (44). Of course this right is limited if there is a contract that obligates the sequenced individual to leave their data on the chain. The right is also limited if the data is anonymized, as it most likely would be on a blockchain. Pseudoanonymized data might still be protected under GDPR. Whether sequence data falls under the concept of anonymized or pseudoanonymized (as it can, apparently be sourced with enough information) will be a limiting factor in the application of the GDPR to genomics on public blockchains because of the immutable nature of the data stored on the blockchain (45). Note however, that Satoshi Nakamoto the pseudoanonymous creator of blockchain technology allowed for the idea of eventually pruning the blockchain of data.

Also, without a central source to contact, there is no data controller who can follow through with the GDPR directives. Although public blockchains could be created to allow for consensus driven implementors of chain governance, including the deletion of GDPR protected data. Immutable chains can deal somewhat with these concerns by storing any truly identifiable information off-chain, allowing those records to be deleted or taken off-line if a GDPR-based request is made.

Private chains can also be designed to allow for changes to the data, albeit at the expense of some of the inherent value associated with using the blockchain technology. Alternatively, private chains can delete the identifier that links the various data sets associated with the sequenced individual if DNA is considered to be fully anonymous. In the example described above, NFTs that are tied to the individuals data can also be pulled from circulation as well, effectively disappearing them from the chain.

## Incentive-based economy

Integrating blockchain technology as a key component in DNA storage enables the user a decentralized and rewarding platform for sharing sensitive data (46). Some might argue that the example of NFTs could be used to create perverse incentives that encourage users to share their genetic and medical information in exchange for an economic reward. This creates an ethical conflict between one's privacy and financial interests (47). Similarly, creating a market to purchase access to data can be seen as counter to the ethos of open science. Although access to data is often purchased in scientific research, creating an actual market may be seen as a step too far. However, the idea that one could profit each time someone accesses your sequence data for research might solve an ongoing concern in genomic research, the possibility of payment may create a counter incentive for those that are often otherwise disincentivized to participate, solving an ongoing concern: the limited representation of minorities in the databases (48). Further, the chilling effect on participation in genomic studies coming from some fearing that their accessible data will be appropriated by the criminal justice system could be countered with the possibility of remuneration.

## Hacking

With blockchains comprising thousands if not millions of interconnected nodes, there is a concern that hackers could target the weakest links to gain access to the entire network. Whereas in a centralized database the owners of the database need only focus on hardening access to a single site, a large network with distributed copies of the data creates significant cybersecurity concerns, especially when all the nodes in the network may not necessarily be under the oversight of the owner of the blockchain as is the case in a public chain. Private chains can minimize the damage of a hack by limiting the amount of information stored on some nodes, leaving most of the valuable information within a centralized location or a handful of reliable nodes. In these cases, whereas a hack targeted at the infrastructure may be just as problematic as a hack in a public chain, it might be less damaging in terms of lost information.

## Biases

In the NFT example described herein, consumers have the ability to select datasets based on desired characteristics of the data and the sequenced individuals. Similarly, the same system was also described as allowing each user to specifically describe the nature of the consent that they were providing for the use of their data. In both instances, there can be concerns that both consumers of data and the sequenced individuals will employ their inherent biases when deciding which demographics they might research or which diseases they will provide consent for, respectively. On paper, this concern could be dealt with through contract; the terms and conditions that could be submitted to both the consumer and the sequenced individual would have to disallow overt biases, although proving actual malice in any perceived biases might be difficult.

## Standards

Databases, regardless as to whether they are distributed or not are made more usable by the standardization of the data that they hold. This usability is increased further with standards that are upheld among many databases. In maintaining a genomic database on the blockchain, standards will also need to be set. Standards are also necessary for the creation of a genomic NFT marketplace. Fortunately, users of blockchains and creators of NFTs are already held to standards that seem to be broadly maintained. With regard to genomic data, a consortium of stakeholders should devise relevant standards for the maintenance of data on and off chains, standards for anonymization and maintaining privacy and standards for reidentification, standards for consent, and standards for interaction with those who have been sequenced. This is non-trivial and could take significantly longer than the creation of the underlying infrastructure.

## Conclusions

We described how an NFT marketplace based on a permissioned private chain could be implemented to minimize many of the ongoing concerns associated with DNA databases. A number of commercial entities have attempted to create various blockchain or blockchain interacting systems as genomic databases. Many are now offline or have not lived up to the hype. In spite of the current crypto-winter, we believe that ultimately some version of a blockchain genomic database will succeed in providing both easy transparent valuable access to genomic researchers while simultaneously providing sequenced individuals with extensive autonomy to both protect their privacy and also profit off of their data.
